# Evaluating putative repellent ‘push’ and attractive ‘pull’ components for manipulating the odour orientation of host-seeking malaria vectors in the peri-domestic space

**DOI:** 10.1186/s13071-020-04556-7

**Published:** 2021-01-11

**Authors:** Margaret Mendi Njoroge, Ulrike Fillinger, Adam Saddler, Sarah Moore, Willem Takken, Joop J. A. van Loon, Alexandra Hiscox

**Affiliations:** 1 International Centre of Insect Physiology and Ecology (icipe), Human Health Theme, Nairobi, 00100 Kenya; 2 Laboratory of Entomology, Wageningen University and Research, P.O. Box 16, 6700 AA Wageningen, The Netherlands; 3 Department of Epidemiology and Public Health, Swiss Tropical and Public Health Institute, Socinstrasse 57, 4051 833 Basel, Switzerland; 4 University of Basel, Petersplatz 1, Basel, Switzerland; 5 Department of Environmental Health and Ecological Sciences, Ifakara Health Institute, P.O. Box 74, Bagamoyo, Tanzania; 6 London School of Hygiene and Tropical Medicine, ARCTEC, Keppel Street, London, WC1E 7HT UK

**Keywords:** Malaria, Vector control, Outdoor-biting, Spatial repellent, PMD, Citriodiol, Transfluthrin, GC-FID, Semi-field study

## Abstract

**Background:**

Novel malaria vector control approaches aim to combine tools for maximum protection. This study aimed to evaluate novel and re-evaluate existing putative repellent ‘push’ and attractive ‘pull’ components for manipulating the odour orientation of malaria vectors in the peri-domestic space.

**Methods:**

*Anopheles arabiensis* outdoor human landing catches and trap comparisons were implemented in large semi-field systems to (i) test the efficacy of Citriodiol^®^ or transfluthrin-treated fabric strips positioned in house eave gaps as push components for preventing bites; (ii) understand the efficacy of MB5-baited Suna-traps in attracting vectors in the presence of a human being; (iii) assess 2-butanone as a CO_2_ replacement for trapping; (iv) determine the protection provided by a full push-pull set up. The air concentrations of the chemical constituents of the push–pull set-up were quantified.

**Results:**

Microencapsulated Citriodiol^®^ eave strips did not provide outdoor protection against host-seeking *An. arabiensis*. Transfluthrin-treated strips reduced the odds of a mosquito landing on the human volunteer (OR 0.17; 95% CI 0.12–0.23). This impact was lower (OR 0.59; 95% CI 0.52–0.66) during the push-pull experiment, which was associated with low nighttime temperatures likely affecting the transfluthrin vaporisation. The MB5-baited Suna trap supplemented with CO_2_ attracted only a third of the released mosquitoes in the absence of a human being; however, with a human volunteer in the same system, the trap caught < 1% of all released mosquitoes. The volunteer consistently attracted over two-thirds of all mosquitoes released. This was the case in the absence (‘pull’ only) and in the presence of a spatial repellent (‘push-pull’), indicating that in its current configuration the tested ‘pull’ does not provide a valuable addition to a spatial repellent. The chemical 2-butanone was ineffective in replacing CO_2_. Transfluthrin was detectable in the air space but with a strong linear reduction in concentrations over 5 m from release. The MB5 constituent chemicals were only irregularly detected, potentially suggesting insufficient release and concentration in the air for attraction.

**Conclusion:**

This step-by-step evaluation of the selected ‘push’ and ‘pull’ components led to a better understanding of their ability to affect host-seeking behaviours of the malaria vector *An. arabiensis* in the peri-domestic space and helps to gauge the impact such tools would have when used in the field for monitoring or control.
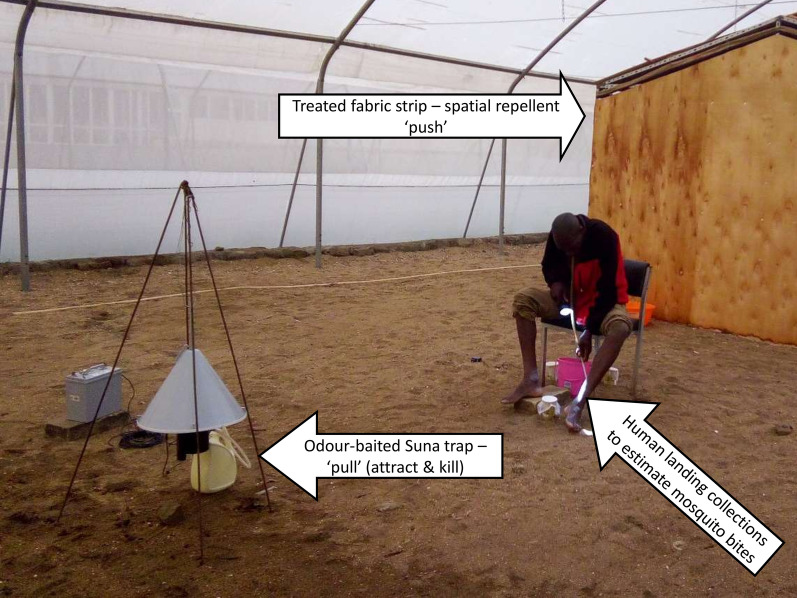

## Background

Despite the impressive efforts made in the past 2 decades, progress in the fight against malaria has stagnated in recent years [1–3]. A large proportion of the reduction in malaria has been attributed to vector control, yet research and operational practice have concentrated on the development of chemotherapy and vaccines, with vector control not expanding its arsenal beyond long-lasting insecticidal nets (LLINs) and indoor residual spraying (IRS) [4]. Increased pyrethroid resistance in malaria vectors [5, 6], shifts in mosquito biting behaviour from predominately endophagic to more exophagic populations [7, 8] and earlier biting [9] demand the re-evaluation of contemporary practices and the development of additional tools addressing current limitations. The World Health Organisation (WHO) endorsed the universal use and application of LLINs and IRS as tools in the fight against malaria [10]. Both of these tools primarily target indoor-biting mosquitoes, which contribute to almost 80% of all malaria transmission [11]. Whilst the remaining outdoor transmission increases in importance once the indoor tools are effectively applied [12, 13], no outdoor tools have been approved by WHO for supplementary mass application [1].

The use of spatial repellents has been proposed to provide protection against bites at a distance from the point of application, which could not only provide potential protection to multiple persons but may also lead to higher compliance due to reduced need for reapplication which is a barrier to effective use of tropical repellents [14–17]. The ability to produce vector-free spaces would make spatial repellents ideal for application in the peri-domestic space, defined as in-and around the outside of the house [14]. Several insecticides already used in public health have, to varying degrees, spatial repellent effects on various mosquito species [18]. These insecticides volatilize more readily than other adulticides and repel, even in instances when the vectors are intrinsically resistant to pyrethroids [19, 20]. One pyrethroid that exhibits spatial repellent properties against mosquitoes at sub-lethal concentrations is transfluthrin [18, 21, 22]. However, in the light of growing pyrethroid resistance it would also be desirable to search for novel active compounds. For example, Citriodiol® sourced from *Eucalyptus citriodora* oil, which includes a minimum 64% para-menthane-3, 8-diol (PMD) as the active ingredient, is used in topical skin repellents [23–25] and has been suggested to have spatial repellent properties [26].

There is a possibility that, when used on their own, spatial repellents might lead to increased biting on unprotected persons through diversion of host-seeking vectors from treated to untreated spaces [27]. To prevent diverted vectors from finding alternative hosts, supplementary tools such as odour-baited traps might be combined with spatial repellents. Odour-baited mass trapping, as a single tool, has been shown to reduce *An. funestus* densities indoors in a recent field trial [28]. Spatial repellents and odour-baited traps target opposing odour-mediated orientations of the mosquito and therefore may work synergistically in a ‘push-pull’ system [26, 29–31].

The term ‘push-pull’ was first conceived as a strategy for insect pest management in Australia in 1987 [32] and the concept is now frequently applied in the control of agricultural pests [33, 34]. The intervention not only offers repulsion from the intended host, but rather redirects them to an alternative that does not lead to disease [31, 33, 35, 36]. An adaptation of this tool for vector control was developed to curb transmission of trypanosomiasis. Cattle provided with a repellent worn on the neck as a push were supplemented with insecticide-treated targets which acted as attractive pull components that killed the flies that landed on them [37]. The reduction in tsetse fly populations was more strongly associated with a combined push-pull set-up than with the repellent and attractant when used separately or not at all [37]. To develop such a ‘push-pull’ strategy for malaria vector control, it is necessary to determine the efficacy of the potential components individually and in combination to understand their contribution to protecting human hosts from bites. The push-pull strategy for malaria vector control targets the odour-mediated orientation of female mosquitoes when searching for a human host and aims to manipulate this behaviour. This requires that effective quantities of the repellent and odour attractants are perceived by the targeted mosquito species within the space that should be protected [38]. Quantification of the airborne concentrations of the chemical constituents of the push-pull control tool might help interpret behavioural responses recorded in bioassays and gauge the influence of weather conditions [38–40]. Such information might inform the spatial arrangement of the push-pull system and assist in identifying needs for improvements of release rates of individual components. Importantly, quantification of chemicals in the air allows for monitoring of safe levels, especially amounts inhaled by humans or levels available to susceptible non-target hosts [40, 41].

This study aimed to evaluate novel and re-evaluate existing, putative repellent ‘push’ and attractive ‘pull’ components for manipulating the odour orientation of malaria vectors in the peri-domestic space with the aim to develop a ‘push-pull’ system that reduces bites and kills vectors. Five objectives were pursued: (i) to test the efficacy of fabric strips treated with either microencapsulated Citriodiol® or with an emulsified concentrate of transfluthrin positioned in open eave gaps on houses as a push component for preventing *Anopheles arabiensis* bites outdoors; (ii) to understand the efficacy of an MB5-blend baited Suna-trap in attracting (pulling) *An. arabiensis* to the trap in the presence of a human being; (iii) to assess the possibility of replacing CO_2_ produced from yeast-sugar fermentation with the putative CO_2_ replacement, 2-butanone, in the Suna trap; (iv) to determine the degree of protection for a human host against mosquito bites by combining push and pull components; (v) to quantify the air concentrations of the chemical constituents of the push-pull mosquito control tool.

## Methods

### Study site

**Fig. 1:**
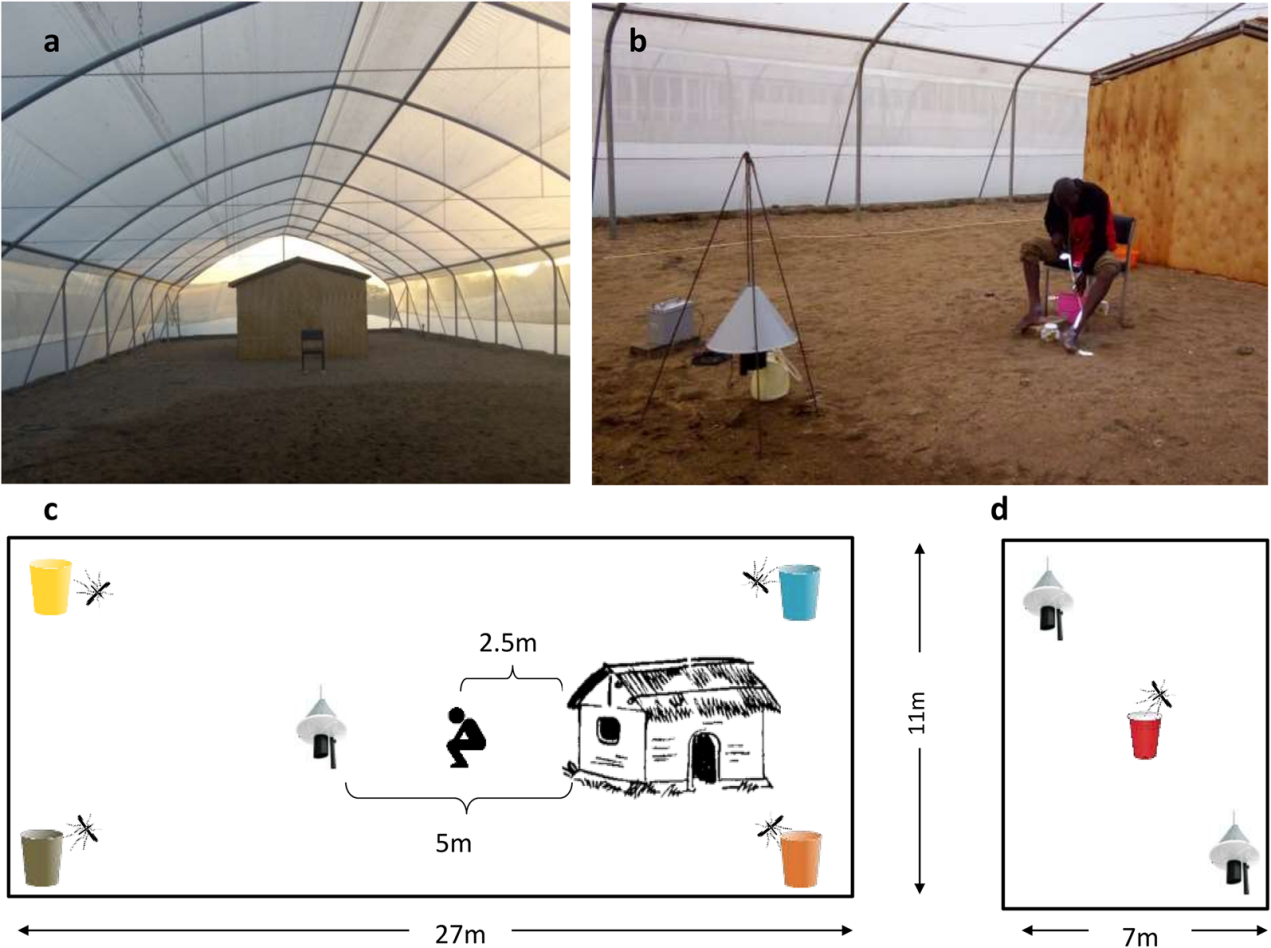
Pictorial presentation of the experimental set-ups in the semi-field systems. View into the large tunnel-shaped semi-field system; 11 m wide and 27 m long (**a**); Volunteer implementing human landing collections between the experimental hut and the Suna trap in the larger system (**b**); schematic description of experiments including HLC outside the hut 2.5 m away from the hut (eave treatments) and the Suna trap. Colour-coded mosquitoes were released from all four corners of the system (**c**); schematic description of experiments in the small semi-field system, 11 m long and 7 m wide, where different trap configurations were tested with two traps included in the system (**d**)

All experiments were carried out in semi-field systems made up of four netting-screened greenhouses located at the International Centre of Insect Physiology and Ecology’s Thomas Odhiambo Campus (*icipe*-TOC) at Mbita, in Homabay County, western Kenya (0°26ʹ06.19″ S, 34°12ʹ53.13″ E; altitude 1,137 m). The majority of experiments (Table 1) were carried out in two large semi-field systems (Amiran Ltd, Nairobi, Kenya) measuring 27 m in length, 11 m in width and 4.3 m at the highest midpoint (Fig. 1).

**Table 1:** Summary of the experiments in relation to research questions

	TEST treatment	CONTROL treatment	Human landing catch	Test and control independent or in competition^a^
Experiment 1				
What is the human biting rate of Anopheles arabiensis released in the semi-field systems between 19.00 and 23.00 h in the absence of any treatment?				
Are the two semi-field systems comparable in the results they generate?				
Are there any differences in catching efficiency/attractiveness of HLC volunteers?				
1.1	No treatment	No treatment	Yes	Independent
Experiment 2				
Can Citriodiol® and/or transfluthrin-treated strips located at eave gaps reduce *An. arabiensis* biting rates compared to untreated controls?				
2.1	1 g/m^2^ microencapsulated Citriodiol^®^	Untreated cotton fabric	Yes	Independent
2.2	11 g/m^2^ microencapsulated Citriodiol^®^	Untreated cotton fabric	Yes	Independent
2.3	1.25 g/m^2^ transfluthrin fabric	Untreated hessian fabric	Yes	Independent
2.4	2.5 g/m^2^ transfluthrin fabric	Untreated hessian fabric	Yes	Independent
Experiment 3				
How effective is the MB5 baited Suna trap in attracting insectary-reared *An. arabiensis* in a large semi-field system in the absence and presence of a human being?				
How does the MB5 cartridge perform in comparison to nylon strips impregnated by investigator?				
Does 2-butanone combined with MB5 perform equally well in attracting insectary-reared *An. arabiensis* in a large semi-field system as CO2 produced from molasses fermentation?				
How does the trapping efficacy compare between Suna traps baited with CO2only and Suna traps baited with the synthetic MB5 lure in addition to CO2?				
3.1	MB5-cartridge baited Suna trap supplemented with CO_2_	MB5-nylon strip baited Suna trap supplemented with CO_2_	No	Competing
3.2	MB5-cartridge baited Suna trap with 2-butanone	MB5-cartridge baited Suna trap with CO_2_	No	Competing
3.3	MB5-cartridge baited Suna trap with 2-butanone	Unbaited Suna trap (no MB5, no CO_2_, only suction fan)	No	Competing
3.4	MB5-cartridge baited Suna trap with CO_2_	Unbaited Suna trap (no MB5) supplemented with CO_2_ only	No	Competing
3.5	MB5-cartridge baited Suna trap supplemented with CO2	Unbaited Suna trap (no MB5, no CO2, only suction fan)	Yes	Independent
3.6	MB5-cartridge baited Suna trap with 2-butanone	Unbaited Suna trap (no MB5, no CO_2_, only suction fan)	Yes	Independent
Experiment 4				
Can Citriodiol® and/or transfluthrin-treated strips located at eave gaps reduce *An. arabiensis* biting rates compared to untreated controls?				
4.1	Transfluthrin 2.5 g/m^2^ eave wrap + MB5-cartridge and with CO_2_	Untreated eave wrap + unbaited Suna trap	Yes	Independent

^a^Two semi-field systems were used for testing test and control treatments independently but concurrently. The treatments were randomly allocated to the two systems. Competing tests were set in the same semi-field system

The two large systems were located in parallel, 10 m apart from each other. The roof covers were made from translucent waterproof Solarig^TM^ material (Amiran Kenya Ltd.) and the sides were made of a 17-mesh netting material (17 apertures per every linear inch (2.54 cm) of mesh). One wooden make-shift hut made from plywood walls attached to angle irons, with grass thatch applied on an open gable roof, was included in each system at opposite ends approximately 5 m away from the shorter walls. The huts were 6.5 m long and 3.5 m wide with a maximum height of 2.5 m (Fig. 1). Between the roof and the walls was a 0.1-m eave gap, a size that was representative of the open eave gaps typical in traditional western Kenyan houses and in other rural African areas [42–44]. The doors and windows of the experimental huts were fully mesh-screened. Mosquitoes could only enter and exit the huts through the eave gaps during experiments.

Few experiments (Table 1) were done in smaller-sized semi-field systems measuring 11 m in length and 7 m in width, with 2.50 m at highest point (Fig. 1). The walls of these were screened with fibreglass netting of the same mesh size as the large systems while the roofs were made of translucent polycarbonate [45]. Ambient temperatures inside the semi-field systems ranged between a minimum of 18°C at night and maximum of 50°C during the day as monitored with data loggers (Tinytag View 2 Gemini data loggers, UK) suspended in the middle of the semi-field systems which recorded temperature readings every 30 min. During the nightly experiments between 19.00 and 23.00 h the average temperature ranged between 21 and 24 °C. The natural floor in all four semi-field systems was covered with a layer of around 20 cm of sand and was watered daily for 2 h, prior to the experiments with free-flying mosquitoes to maintain a relative humidity in the systems of around 70%. A summary of all experiments is found in Table 1. All experiments with human landing volunteers included were implemented in the large semi-field systems.

### Mosquitoes

All experiments were implemented with host-seeking females of *An. Arabiensis* Mbita strain, aged between 3 and 5 days post-emergence. Mosquitoes were reared under ambient conditions at *icipe*-TOC following standard operating procedures [46].Nulliparous mosquitoes that had not taken a blood-meal previously were activated to host-seek by placing a human hand near the outside of the mosquito cage and only those that responded to human odours were aspirated and used in experiments. In experiments including a human volunteer, 160 females were released in each semi-field system per experimental night. In experiments including traps only, 200 females were released in each system per night. The mosquitoes were transferred from rearing cages into release cups using mouth aspirators. In the release cups they were starved from water and glucose for a minimum of 3 and a maximum of 5 h prior to release. Anticipating that the orientation of a female mosquito in the system will be affected by the direction of air movement, obstructions like the hut and outside light sources, mosquitoes were released from cups in all four corners of a semi-field system to account for such factors. In experiments including a human, each of the four release cups contained 40 *An. arabiensis* females (total = 160 females). Mosquitoes in each release cup were dusted using a distinct colour of fluorescent dye to distinguish them according to the four corners of release [47]. In choice experiments with traps only, the traps were rotated through the corners of the semi-field system and the mosquitoes released from one release cup in the centre of the screen house.

### Repellent-treated fabrics (push component)

A passive release mechanism for spatial repellents was favoured in this project in order to reduce the operational complexity that would come with an electricity-powered active dispenser. Hence, the two test compounds, Citriodiol^®^ and transfluthrin, were both presented on fabrics which can be easily attached to open eave gaps on houses [30].

Citriodiol^®^ (Citrefine International Ltd) was microencapsulated by Devan Chemicals, Portugal and applied to fabric by Utexbel, Belgium, using the solvent evaporation technique with poly lactic acid as a shell material as previously described [29, 48]. The fabric was shipped to Kenya and stored in a cold and dark room prior to use. Two fabric weights with two loads of Citriodiol^®^ were tested. The first was a 100% cotton fabric (65 g/m^2^) with 1 g/m^2^ Citriodiol^®^ and the second had a fabric weight of 550 g/m^2^ with a Citriodiol^®^ load of 11 g/m^2^ (microcapsules for both were 15 μm with 17% weight of the active ingredient of para menthane-3, 8-diol; PMD).

Transfluthrin (Bayer Global, Leverkusen, Germany) was obtained as an emulsified concentrate (EC) of 0.2 g/ml and applied on hessian fabric (obtained as burlap material from local markets in Kenya) to achieve two final loads on the fabric, namely 1.25 and 2.5 g/m^2^ [49]. The impregnation of the hessian fabric was done in the laboratory at *icipe*-TOC where the respective amount of transfluthrin EC was added into to water that was sufficient for wetting the entire length of fabric without any water remaining. The fabric was soaked well and dried in the shade overnight and then wrapped up in aluminium foil and stored in a cold (4 °C) and dark room prior to use.

The treated fabrics were cut into strips measuring 21 m long, corresponding to the perimeter of the eave gaps of the experimental huts, and a width of 0.05 m, corresponding to half of the width of the eave gap. Correspondingly, untreated fabric strips were prepared in the same dimensions and used for the control experiments. The fabric strips were fixed half an hour prior to mosquito release with flexible aluminium wires in such a way that they were covering only part of the eave gap leaving a similar space above and below (2.5 cm each) to allow for movement of air. They represented an incomplete, easy to fix fabric strip along the gaps, not an eave screen. The fabric strips were removed in the morning and stored in the cold room till the next experimental night. Fabric strips were used continuously for a maximum of eight experimental nights. Experiments were done for 16 nights; hence two strips were used per experiment.

### Suna trap and odour lure (pull component)

Odour-baited Suna traps were used throughout as pull devices. The trap’s development, appearance and operation are described in detail elsewhere [50].The principle odour bait re-evaluated in experiments was a synthetic chemical blend aiming to mimic human host odours and has previously been published under the name ‘Mbita Blend 5’ or MB5 (51, 52). The MB5 comprised ammonia (2.5% in water), l-(+)-lactic acid (85% in water), tetradecanoic acid (0.00025 g/l in ethanol), 3-methyl-1-butanol (0.000001% in water) and butan-1-amine, prepared at a concentration of 0.001% in paraffin oil [26], and was recently associated with significant reductions in *An. funestus* populations during a mass-trapping vector control trial [28]. Two dispensing substrates of MB5 were compared. As in previously published work, MB5 was presented on nylon strips [53, 54] where each strip was treated with one chemical of the blend and consequently five strips inserted in the trap. This was compared to a novel, slow-release cartridge developed by Biogents (Biogents Cartridge Lure (Mosquito Attractant) -LI-MR-43, Regensburg, Germany) containing the same five chemicals.

Carbon dioxide has been repeatedly reported as essential in combination with an odour blend for attracting host-seeking malaria vectors [55–57] and remains one of the most challenging obstacles to area-wide operational use of odour-baited traps. Carbon dioxide gas released from cylinders is not manageable under field conditions; hence, a previously developed method of producing CO_2_ from fermenting sugar or molasses solution using yeast is now widely used [58–60]. However, the amount of sugar or molasses needed for every trap night is still prohibitive for operational vector control. The chemical 2-butanone has been proposed as a CO_2_ mimic for several species of mosquitoes based on electrophysiological assays and activation patterns of CO^2^-detecting neurons; however, experimental trapping data with *Anopheles* mosquitoes has led to controversial results under different experimental settings [61, 62]. Here, CO_2_ from fermentation was compared with 2-butanone-treated (0.1 ml) nylon strips [62] to gain a better understanding of its effectiveness as a supplement of the odour bait in a Suna trap for reducing *An. arabiensis* bites.

In experiments including a human volunteer, a single odour-baited Suna trap was positioned 5 m from the experimental hut, with the volunteer seated mid-way between the hut and the trap in a straight transect (Fig. 1c). The trap was suspended above the ground using a tripod [50, 62] with the main odour-release point, which is the bottom of the funnel, approximately 0.3 m off the ground. In experiments without a human volunteer, two traps were positioned at diagonally opposite corners of the small semi-field system approximately 13 m from each other and less than 1 m from the walls of the system (Fig. 1d).

### Estimation of vector landing rates

Human landing catches (HLC) were carried out as the primary outcome measurement and were conducted on a randomly rotating basis by four adult men (aged between 18 and 50 years) seated 2.5 m away from the experimental hut to mimic outdoor biting in a natural setting where people would spend time outside the house during the evening hours. Two volunteers were required per night. In preparation of the experiments, they cleaned their feet and lower legs with odourless soap and took position on a chair as shown in Fig. 1. Collections were done for 4 h from 19.00 to 23.00 h, with volunteers mouth-aspirating host-seeking *An. arabiensis* females as soon as they landed on their lower legs [63]. The mosquitoes were transferred to collection cups, separated hourly. Protective jackets and shoes were worn to protect heads, arms and feet against bites and torches were used for visualization of mosquitoes when aspirating. Volunteers were randomly allotted to the semi-field system and the experiment therein to overcome potential confounding due to differences in collection efficacy and individual attractiveness to mosquitoes.

### Experimental procedures

All experiments and their guiding research questions are detailed in Table 1. Those including human landing catches were conducted as set in two semi-field systems concurrently. All experiments were replicated over 16 nights. A baseline experiment was conducted to understand the mosquito response rate to human volunteers in the two semi-field systems in the absence of any behaviour modulating chemicals. This provided a reference for other experimental sets and helped gauge any differences in attractiveness and catching efficiency of the volunteers or between the two semi-field systems. This experiment also helped to understand the response rates that can be expected from receptive host-seeking mosquitoes in the system. Following this, a threshold was established where, if the response rate in the presence of a human volunteer was < 50% in the control treatment, results were discarded and the replicate repeated. Spatial repellent treatments were rotated weekly given the need to air between treatments to avoid cross-contamination. Experiments were done for 4 consecutive nights and then all test devices and chemical odours were withdrawn from the semi-field systems for 3 days. In the following week, the treatments were crossed over between the semi-field systems. Trap-only experiments were conducted through the night from 19.00 to 07.00 h the next morning.

### Simulation-based power analysis

A simulation-based power analysis [64] was implemented for a 2 × 2 Latin square experiment with two treatments each tested by four volunteers in two semi-field systems. The aim was to be able to measure a 50% reduction in human landing rate; hence, a recapture rate of 60% in the control and 30% in the push-pull experiment was used for the estimation. Assuming 160 mosquitoes released in each semi-field system, and assuming 10% dispersion due to variability between the semi-field systems, 10% variations between mosquito releases and 50% variability between the HLC volunteers, 1000 simulations resulted in an estimated power of 0.94 (95% CI 0.87–0.98) to detect a 50% reduction in human landing rate for 16 replications.

### Air sampling and detection of volatile chemicals released by the push-pull components

Air was sampled in one of the large semi-field systems in the presence of a fully set push-pull system, consisting of 2.5 g/m^2^ transfluthrin fabric strips on eave gaps and a Suna trap baited with MB5 nylon strips and CO_2_ generated through fermentation of molasses. Air was pumped through adsorbent Tenax traps (30 mg; GERSTEL-Twister Desorption glass liners from GERSTEL, Muelheim an der Ruhr, Germany, glass wool from Supelco, Bellefonte, PA, USA, and 25 mg of Tenax^**®**^ TA polymer 60–80 mesh from Supelco, Bellefonte, PA, USA). Micro-diaphragm gas pumps were used at the rate of 400 ml/min resulting in a total of 120 l of air passing through each trap over a 5-h sampling period (18.00–23.00 h), chosen to align with the time period when human landing catches were implemented under experimental conditions. The air sampling was carried out in the absence of a human to focus on the chemicals released by the push-pull components. All chemicals collected were reported as concentrations averaged over the time-period of trapping and calculated as nanograms per litre of air sampled; subsequently referred to only as ‘concentration’ in ng/l.

**Fig. 2:**
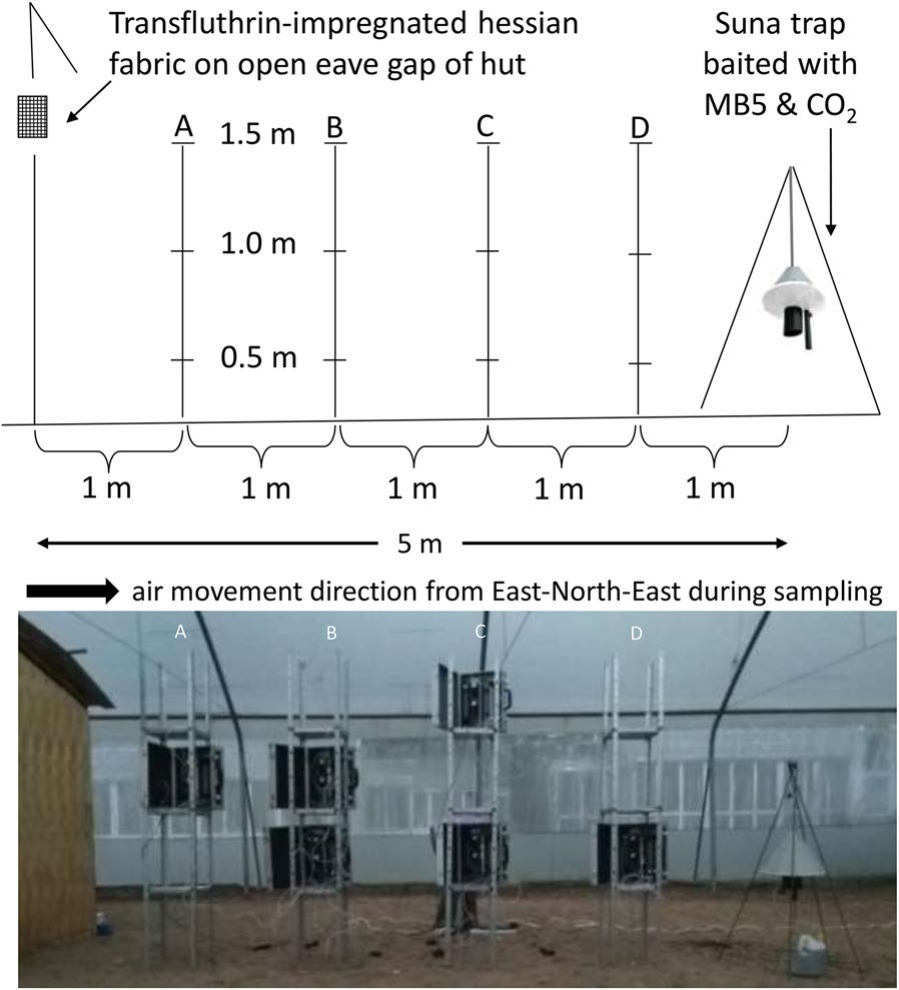
Pictorial presentation of air sampling set-up. Air-entrainment pumps were positioned at 1 m (**a**), 2 m (**b**), 3 m (**c**) and 4 m (**d**) distance from the experimental hut where a transfluthrin-treated fabric was positioned at the eave gap. The baited Suna trap was 5 m away from the hut. Sampling was done at every position at 3 heights: 0.5, 1.0 and 1.5 m above the ground

Twelve locations were sampled in a transect between the transfluthrin-treated fabric at the experimental hut and the odour-baited Suna trap placed at a distance of 5 m away from the hut (Fig. 2).

Sampling was done every 1 m between the fabric (house wall) and the trap, at four distances. At every distance, sampling was done at three heights: 0.5, 1.0 and 1.5 m (Fig. 2). Sampling was replicated over 5 non-consecutive days, with each set-up using freshly treated eave fabric and new nylon strips for the odour blend to ensure consistency in the initial concentrations. At the conclusion of each sampling event, adsorbent filters were stored at -80°C until chemical analysis. For quantification, trapped volatiles were eluted using dichloromethane (DCM; CAS 75-09-2, Merck, Massachusetts, USA) and analysed using gas chromatography (GC) with flame-ionization detection(GC-FID; Agilent 7890B, Agilent Technologies, California, USA; (65)). The lowest detection temperature was set at 35 °C and the highest was set at 280 °C. A Solgewax (SGE, Australia) column, 30 m long and 0.25 mm in diameter with an internal diameter of 0.2 µm, was used.

To obtain calibration lines for quantification of air concentrations for all the push-pull constituent chemicals (except l-lactic acid and ammonia solution) concentration gradients were obtained by preparing dilutions of the chemicals from the stock solutions ranging from 0.5 to 10 ng/µl resulting in the preparation of the following concentrations: 0.5, 1.0, 2.0, 4.0, 6, 8 and 10 µg/µl. Each concentration was injected separately into the GC-FID, and then the area under the curve determined and plotted against the concentration. A linear equation was obtained by plotting all the concentrations against all the areas of each chemical where y represented the area under the curve while x represented the concentration in nanograms per litre of air. All linear equations met the minimum qualification of *R*^2^ value of 0.98. Subsequent determination of concentration was determined by obtaining the area under the curve directly from the GC-FID and solving for *x* in the linear equation of each chemical [66–68].

To determine the direction and strength of air movements as well as temperature during collections, a long-range wireless wind logger (Navis WL 11X, NAVIS Elektronika, Kamnik, Slovenia) was set up at 2 m height next to the Suna trap during the air sampling period. Logging of parameters was done in 5-min intervals.

### Data analysis

Analyses of experimental data were done using R version 3.5.1[69]. Data were descriptively explored and presented by generating box plots, where the boundary of the box closest to zero indicates the 25th percentile, the black line within the box marks the median and the boundary of the box farthest from zero the 75th percentile. Whiskers above and below the box indicate the 10th and 90th percentiles. All mosquito catches were analysed as proportions (number of mosquitoes attempting to bite either out of the total number released in the system or out of the total number recollected with HLCs and/or traps) using generalized linear mixed models (lme4) with the experimental night and HLC volunteer (where applicable) included as random factors. All proportions were modelled using binomial probability distributions with logit link functions fitted. Treatment group was included as the fixed factor in the models with the control group as reference. The semi-field system ID was also included as factor and retained in the final model only if significantly associated with the outcome. Where applicable, interactions were explored. All analyses of volatile chemicals in air samples were done by calculating the means and the standard error for measurements made at every position across the 5 sampling days. Analysis of variance to determine differences between sampling positions A–D and sampling heights 0.5–1.5 m were done for each chemical detected.

## Results

### Experiment 1: establishing landing rates of *An. arabiensis* in semi-field systems in the absence of treatments

On average, 67% (95% CI 62–72%) of the released mosquitoes were recaptured in semi-field system A and 62% (95% CI 56–67%) in system B within 4 h of human landing collections (19.00–23.00 h) with volunteers seated 2.5 m away from the hut. Adjusting for time of collection and volunteer, the semi-field system (A or B) was not associated with the odds of recapturing a mosquito (OR 0.96, *p* = 0.286; Table 2).

**Table 2:** Association between outdoor *An. arabiensis* landing and time of collection, semi-field system and volunteer

Explanatory variables in multivariable analysis	Odds ratio (OR)	Confidence interval (CI)		*p*-value
		Lower CI	Higher CI	
Collection time^a^				
19.00–20.00 h	1			
20.00–21.00 h	0.47	0.426	0.511	< 0.001
21.00–22.00 h	0.28	0.250	0.307	< 0.001
Semi-field system ID				
A	1			
B	0.96	0.868	1.043	0.286
HLC volunteer ID				
No. 1	1			
No. 2	1.25	1.098	1.425	< 0.001
No. 3	1.26	1.099	1.439	< 0.001
No. 4	1.17	1.018	1.338	0.027

^a^No mosquitoes were captured between 22.00 and 23.00 h; hence, this category was not included in analysis

**Fig. 3:**
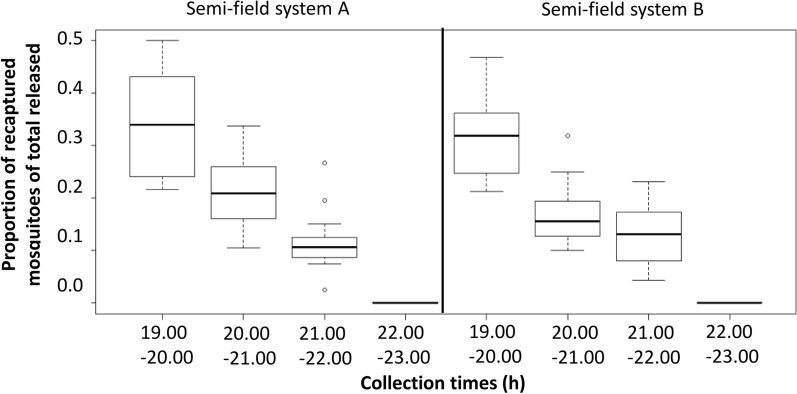
Hourly *Anopheles arabiensis* landing on a human volunteer in two semi-field systems in the absence of any test. Based on human landing collections by four volunteers that were randomly rotated between the systems; out of all mosquitoes released (*n* = 160/experimental night)

Collection time, however, was significantly associated with the outcome (Fig. 3). The largest proportion of host-seeking mosquitoes was recaptured in the first hour, whilst none were captured in the fourth collection hour (22.00–23.00 h). The odds of collecting a landing mosquito decreased over time (Table 2, Fig. 3).

There was some variability in the collection efficiency between volunteers either due to attractiveness or skills, with one of the volunteers collecting fewer mosquitoes than the others (Table 2). Based on this, in consecutive analyses, the volunteer IDs were included as a random factor in the model. Host-seeking females were recaptured in similar proportions from all four release corners. There was no significant association between the human landing rate and temperature or relative humidity in the semi-field system during experimental nights.

These results, obtained in the absence of any treatment, confirmed that the insectary-reared *An. arabiensis* were highly responsive to a human blood host and that both semi-field systems supported reproducible results in the presence of a human volunteer. Subsequently, this set of experiments served as a reference for all following experiments with various treatments included.

### Experiment 2: investigating potential push components for a push-pull vector control strategy

#### Microencapsulated Citriodiol^***®***^ fabric strips on open eave gaps

**Fig. 4:**
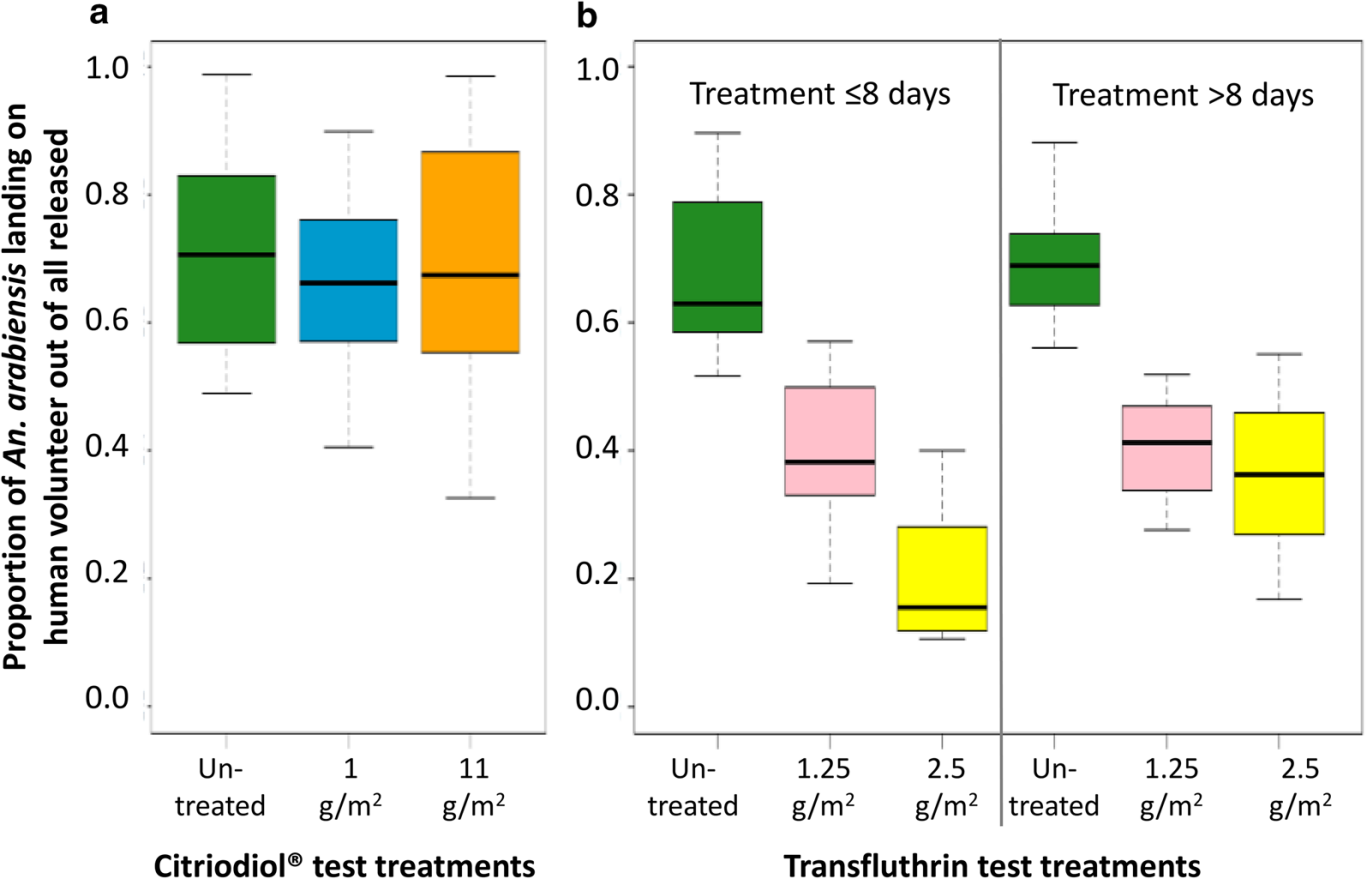
*Anopheles arabiensis* host-seeking while exposed to putative spatial repellents in semi-field systems as estimated with HLC. Two formulations of microencapsulated Citriodiol (1 and 11 g/m^2^) were tested compared to untreated control fabric (**a**). Two impregnation concentrations of transfluthrin were tested on hessian fabric, 1.25 and 2.5 g/m^2^, compared to untreated control fabric (**b**). The proportions are based on the total number of mosquitoes released (*n* = 160/experimental night)

Neither the eave fabric encapsulated with 1 g/m^2^ Citriodiol^®^ (*p* = 0.488) nor the heavier fabric with 11 g/m^2^ Citriodiol^®^ (*p* = 0.633) were associated with a reduction in the proportion of mosquitoes landing on a volunteer when compared to untreated controls (Fig. 4a). In all experimental treatments, catches were similar and consistent, ranging between a median of 66–71% of all released mosquitoes recovered through HLC.

### Transfluthrin EC-impregnated hessian fabric strips on open eave gaps

Transfluthrin-treated fabric strips, at both treatment loads, were significantly associated with reduced human landing at a distance of 2.5 m away from the hut (Fig. 4b, Table 3). The odds of a mosquito landing on the volunteer in the presence of the 1.25 g/m^2^ transfluthrin fabric were decreased by a factor of 2.5 (OR 0.39; Table 3) compared to the odds in the presence of the untreated control fabric. This was consistent over time, even when the fabric used in the experiment had been treated over 8 days prior. The higher load of 2.5 g/m^2^ resulted in a significantly higher protection with the odds of a mosquito landing decreased by a factor of 16 (OR 0.06; Table 3) compared to the odds of landing in the control. However, this protection reduced when the age of the treated fabric increased. When the fabric treatment had been done more than a week prior to testing, the odds of receiving a bite increased nearly threefold compared to the fresh treatment (Table 3) but was still superior to the lower load. The median percentage of 63–70% of released mosquitoes landing on the HLC volunteer in the experiments with untreated fabric related well to the reference experiment without any treatments included and confirmed the reproducibility of the test system.

**Table 3:** Association between outdoor *An. arabiensis* landing and transfluthrin-treated fabrics (1.25 and 2.5 g/m^2^) around eave gaps

Explanatory variables in multivariable analysis^a^	Odds ratio (OR)	Confidence interval (CI)		*p* value
		Lower CI	Higher CI	
Transfluthrin concentration on fabric strip placed on open eave gap of hut				
Untreated	1			
1.25 g/m^2^	0.39	0.34	0.45	< 0.001
2.5 g/m^2^	0.06	0.05	0.08	< 0.001
Time post-treatment				
< 8 days	1			
> 8 days	1.22	0.82	1.81	0.325
Interaction between transfluthrin concentration × time post-treatment				
1.25 g/m^2^ × <8 days	1			
2.5 g/m^2^ × <8 days	1			
1.25 g/m^2^ × >8 days	1.07	0.81	1.40	0.641
2.5 g/m^2^ × >8 days	2.78	2.02	3.82	< 0.001

^a^Human landing collections were done nightly for 4 h (19.00–23.00 h).

### Experiment 3: investigating pull components for a push-pull vector control strategy

#### Comparing the attractiveness of two odour-dispensing substrates for use in Suna traps

Previously, the MB5 blend was prepared experimentally by manually treating nylon strips with the five chemicals [26, 53, 54]. For operational large-scale use, this would not be a feasible method; hence, a commercial cartridge was developed (Biogents, Germany) that would be easy to use and replace by lay personnel. The competitiveness of the cartridge in attracting mosquitoes to the trap was tested by comparing it to a trap with treated nylon strips in the same small, semi-field system in the absence of a human volunteer. In addition to the chemical blend, CO_2_ was released in both traps during experiments using the fermentation method [51, 70].

**Fig. 5:**
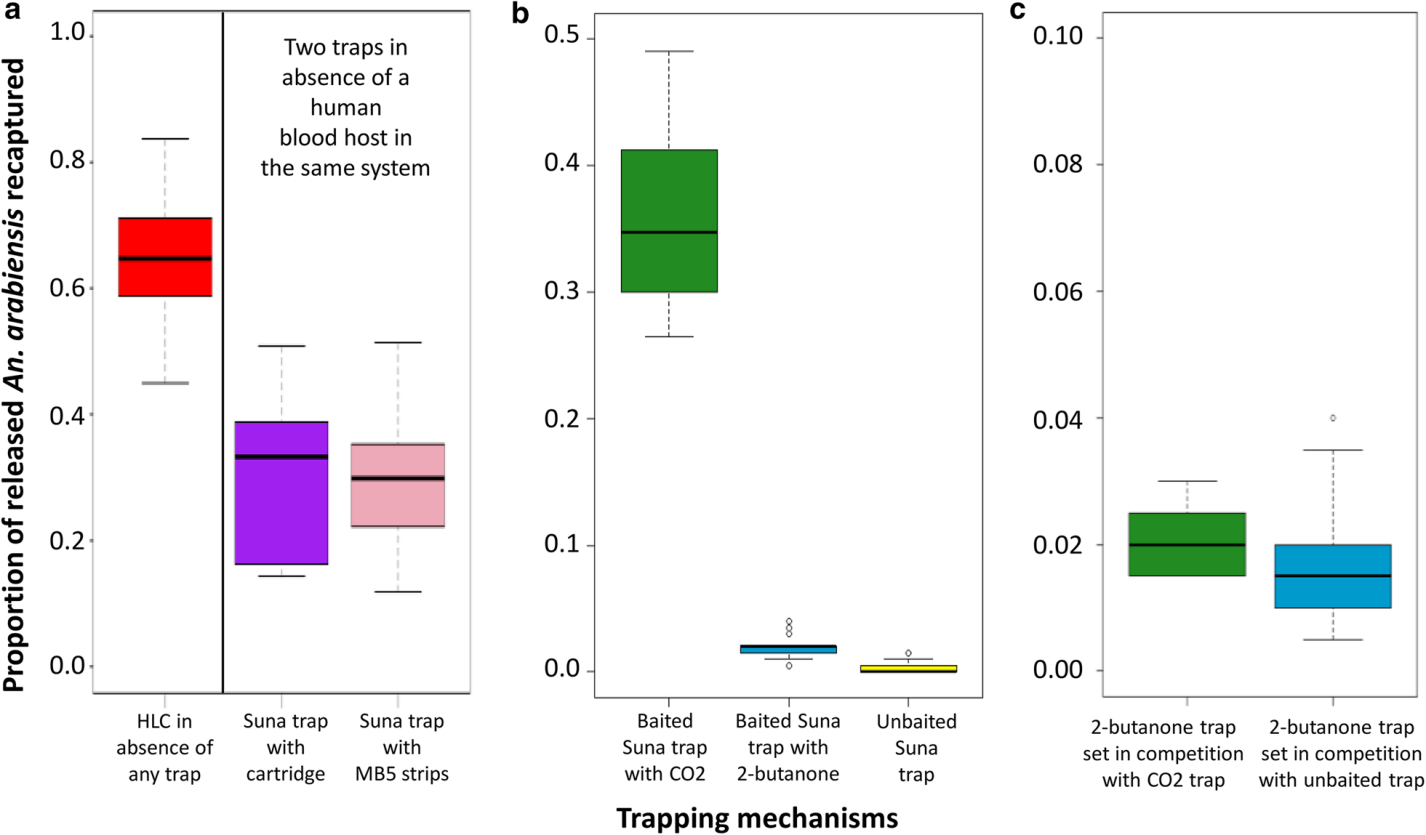
Exploration of the impact of a novel MB5-release cartridge instead of treated nylon strips and 2-butanone instead of CO_2_ on the *An. arabiensis* trapping efficiency of Suna traps under semi-field conditions. **a** Compares the attractiveness of Suna traps baited either with MB5 treated nylon strips or MB5 containing cartridges (Biogents, Germany), both supplemented with CO_2_; the attractiveness of a human is shown as reference. **b** Evaluates the effectiveness of 2-butanone as a CO_2_ replacement for supplementation of MB5-baited Suna traps for attraction of *An. arabiensis.* The box plots in **c** compare the proportion of mosquitoes recaptured with 2-butanone supplemented traps when tested in choice tests**.** Note the different scales of the Y-axes in the figures. A total of 200 host-seeking mosquitoes were released per experimental night. The traps were run overnight for 12 h

The CO_2_-supplemented Suna traps were equally efficient in recapturing host-seeking *An. arabiensis* females released in semi-field systems, irrespective of the presentation of the chemical blend on nylon strips or enclosed in a slow-release cartridge. The two traps together recaptured 61% (95% CI 55–67%) of the released mosquitoes, with a balanced 1:1 distribution (approximately 30% in a single trap) between the two types of blend dispensers (Fig. 5a). Of all trapped females, 49% (95% CI 41–58%) were collected with the cartridge-baited trap. Since there was no advantage of using treated nylon strips, the cartridge was used for all further experiments.

### Exploring the effectiveness of replacing CO_***2***_ with 2-butanone as supplement in MB5-baited Suna traps for the attraction of host-seeking * An. arabiensis*

Experiments were implemented in the absence of a human volunteer with traps set up in competition. There was a strong association between the proportion of mosquitoes recaptured and the test (CO_2_, 2-butanone or nothing). The odds of catching a mosquito in an MB5-baited trap supplemented with 2-butanone were by a factor of 30 lower than the odds of catching a mosquito if the trap was baited with CO_2_ from fermentation (Table 4). The CO_2_-supplemented trap recaptured around 36% (95% CI 32–39%) of the released *An. arabiensis* females whilst the 2-butanone supplemented trap and the unbaited trap without any supplement recaptured well below 0.5% of the released mosquitoes (Fig. 5b). The low catching efficiency of an MB5-baited Suna trap supplemented with 2-butanone was similar in choice tests where the 2-butanone trap was tested in presence of a CO_2_ trap and where the 2-butanone trap was tested in the presence of a completely unbaited trap (*p* = 0.337; Fig. 5c). Notably, the attraction of an odour-baited Suna trap appears to be largely due to the inclusion of only fermentation-based CO_2._ The chemical blend (MB5) added very little (CO_2_ only vs. reference of MB5 plus CO_2_: OR 0.73; 95% CI 0.64–0.84; Table 4) to the attraction of host-seeking *An. arabiensis*.

**Table 4:** Model outputs for experiments aiming to investigate the performance of 2-butanone and CO_2_ in Suna traps (a) and to evaluate the Suna trap in presence of a human blood host (b)

Explanatory variables	Odds ratio (OR)	Confidence interval (CI)		*p*-value
		Lower CI	Higher CI	
a. Exploring the association between 2-butanone or CO_2_ supplement to Suna traps and the proportion of released *An. arabiensis* trapped				
MB5-cartridge baited Suna trap supplemented with CO_2_	1			
MB5-cartridge baited Suna trap supplemented with 2-butanone	0.03	0.03	0.04	< 0.001
Unbaited Suna trap (no MB5, no CO_2_, only suction fan)	0.01	0.00	0.01	< 0.001
Suna trap baited with CO_2_ only (no MB5)	0.73	0.64	0.84	< 0.001
b. Exploring the association between the proportion of released *An. arabiensis* recaptured by human landing volunteers and the presence of a pull device				
HLC in presence of MB5-baited Suna trap and CO_2_	1			
HLC in presence of MB5-baited Suna trap and 2-butanone	0.95	0.86	1.06	0.374
HLC in presence of an unbaited Suna trap unbaited fan	1.06	0.98	1.15	0.170

### Testing the effectiveness of MB5-baited Suna traps as pull devices for trapping *An. arabiensis* in close vicinity of a human blood host

**Fig. 6:**
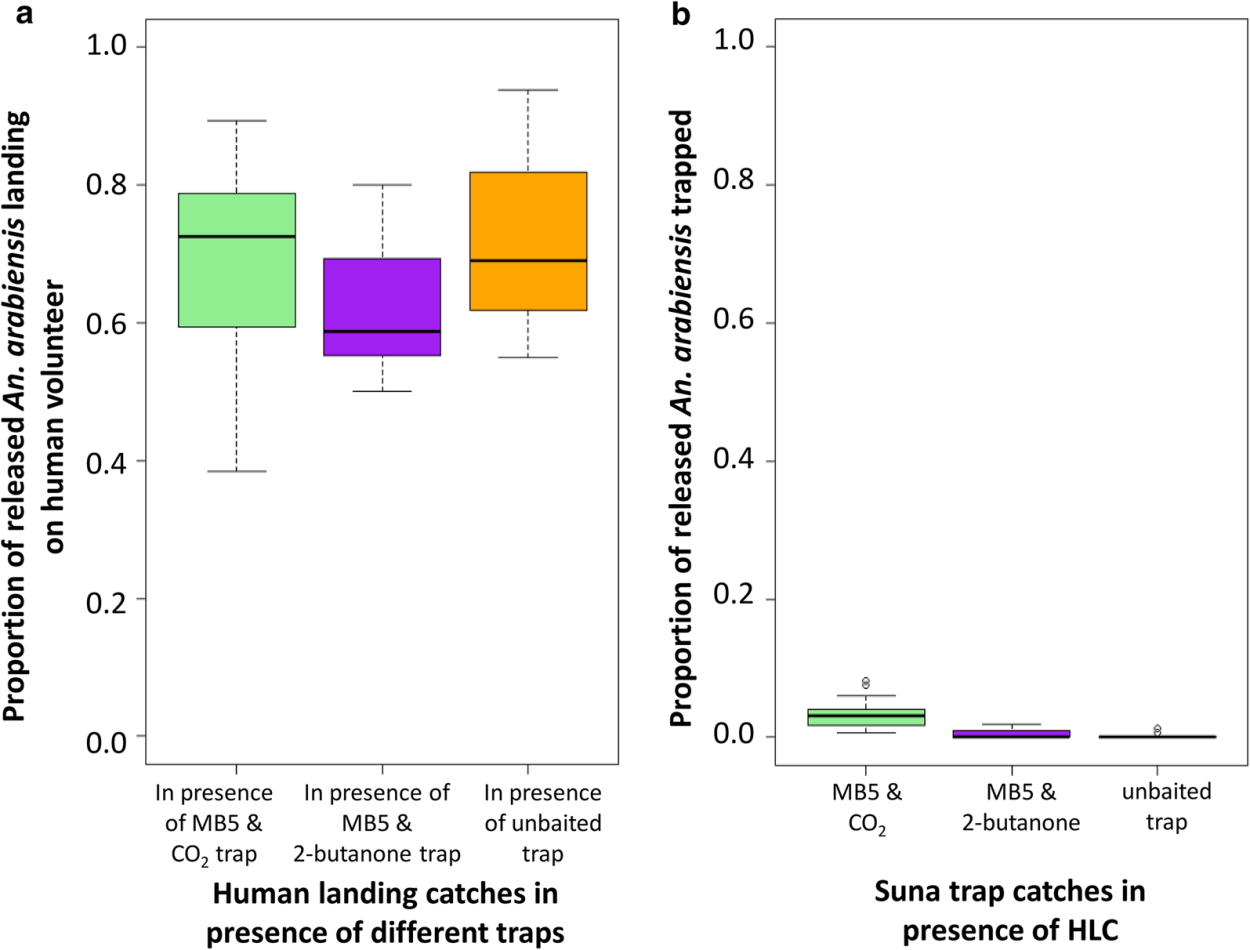
Exploring the attractiveness of MB5-baited Suna traps as pull devices for trapping *An. arabiensis* in close vicinity of a human blood host. **a** The attraction of mosquitoes to human landing volunteers is explored in the presence of Suna traps that are either baited with MB5 and CO_2_ or with MB5 and 2-butanone or are unbaited with only the fan running. **b** Attraction of mosquitoes to the three different traps in the presence of the human blood host is compared. A total of 160 host-seeking mosquitoes were released in the semi-field system per experimental night. HLC was done for 4 h (19.00–23.00 h) and the traps ran overnight for 12 h

Neither of the MB5-baited traps, either supplemented with CO_2_ or with 2-butanone, performed well in the presence of a human blood host (Fig. 6). Whilst in the absence of a human, the MB5-baited Suna trap supplemented with CO_2_ recaptured at least half of what was recaptured by a human volunteer (Fig. 5a), hardly any host-seeking *An. arabiensis* were trapped in the presence of a human blood host (Fig. 6).

A total of 6901 host-seeking mosquitoes were collected by HLC and traps over all experimental nights, out of which only 1.5% (*n *= 103) were trapped in the Suna traps, whilst the remaining 98.5% were attracted to the human landing volunteers. Consequently, the proportion of host-seeking mosquitoes landing on the volunteer was not affected by the presence of a trap in the system (Fig. 6a). The proportions landing were equally high in systems where there was only an unbaited trap, in systems where the trap was baited with MB5 and 2-butanone as well as in systems where the trap was baited with MB5 and CO_2_ from sugar fermentation (Table 4).

### Experiment 4: investigating the impact of a complete push-pull set-up

The push system consisting of the 2.5 g/m^2^ transfluthrin-impregnated hessian fabric placed around the eave gaps of the experimental hut was combined with the MB5-baited Suna trap supplemented with CO_2_. This was the only pull treatment that was effective in attracting mosquitoes in the absence of a human. The combination was tested since it was considered plausible that the spatial repellent might mask the human odour and hence the trap serving as pull might be more effective than when tested in the presence of a human without the push component.

**Fig. 7:**
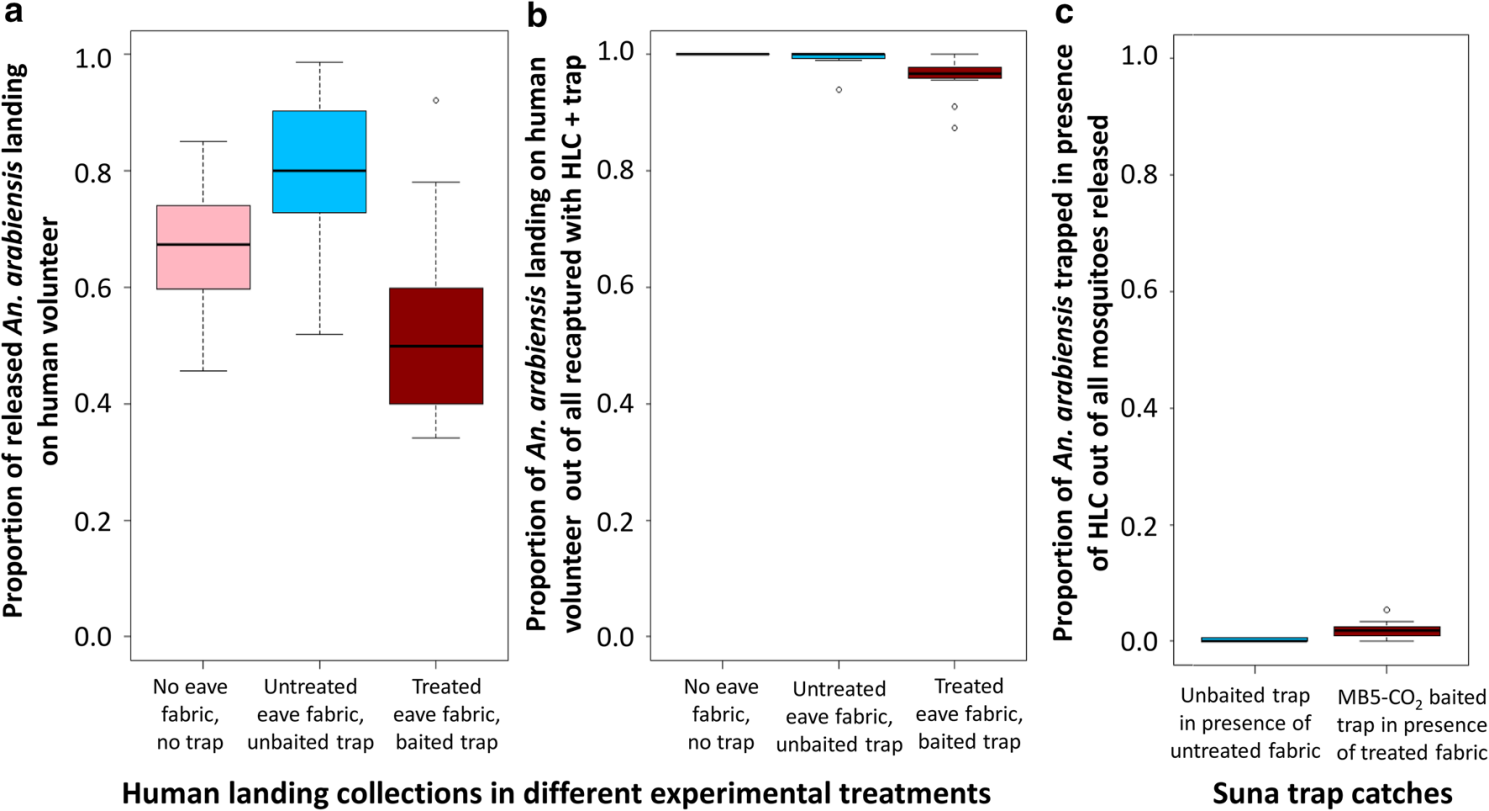
Exploring the impact of a complete push-pull set-up on *An. arabiensis* landing on a human volunteer or being attracted to a trap. Proportion of released mosquitoes landing on the human volunteer outside the hut in the presence of a Suna trap (**a**). Proportion of mosquitoes collected while landing on the human volunteer out of all mosquitoes recollected (total of HLC and trap catches) (**b**). Proportion of mosquitoes trapped in unbaited or baited Suna traps (in presence of HLC) out of all mosquitoes released (**c**). A total of 160 host-seeking mosquitoes were released in the semi-field system per experimental night. HLCs were done for 4 h (19.00–23.00 h) and the traps ran overnight for 12 h

Comparing the functional push-pull set-up with the set-up containing all components but without chemicals (untreated), the odds of receiving a mosquito landing to bite were reduced by a factor of 3.4 in the presence of the push-pull system (OR 0.29 (95% CI 0.25–0.34), *p* < 0.001; Fig. 7). However, this result needs to be interpreted with caution since the reference set-up here (all components without chemicals) already presents an intervention which was associated with increased outdoor biting compared to the control where all components were absent. The presence of untreated and unbaited components was associated with a higher odds of a mosquito landing on a volunteer (OR 2.22 (95% CI 1.96–2.52), *p* < 0.001) compared to the setting where all components were completely absent. It is unclear if this might be due to the fabric strips preventing mosquitoes from entering the hut, hence keeping them closer to the human, or if the observation might be due to other unaccounted conditions given that the two control experiments were implemented at different time points (Fig. 7). A more conservative approach in estimating the impact is therefore to compare the odds of a mosquito trying to bite a human volunteer between the push-pull system and the control without any intervention. In this case, the odds were reduced by a factor of 1.7 in the presence of the repellent-treated and odour-baited push-pull system (OR 0.59 (95% CI 0.52–0.66), *p* < 0.001) compared to the control. Notably, the estimated reduction in the proportion of *An. arabiensis* landing on the human host in this combined push-pull experiment was much lower than it was in experiment 2 when the push was tested alone (2.5 g/m^2^transfluthrin fabric compared to untreated fabric OR 0.17 after 1 week post-treatment).

### Temperature and relative humidity variations during experiments

**Fig. 8:**
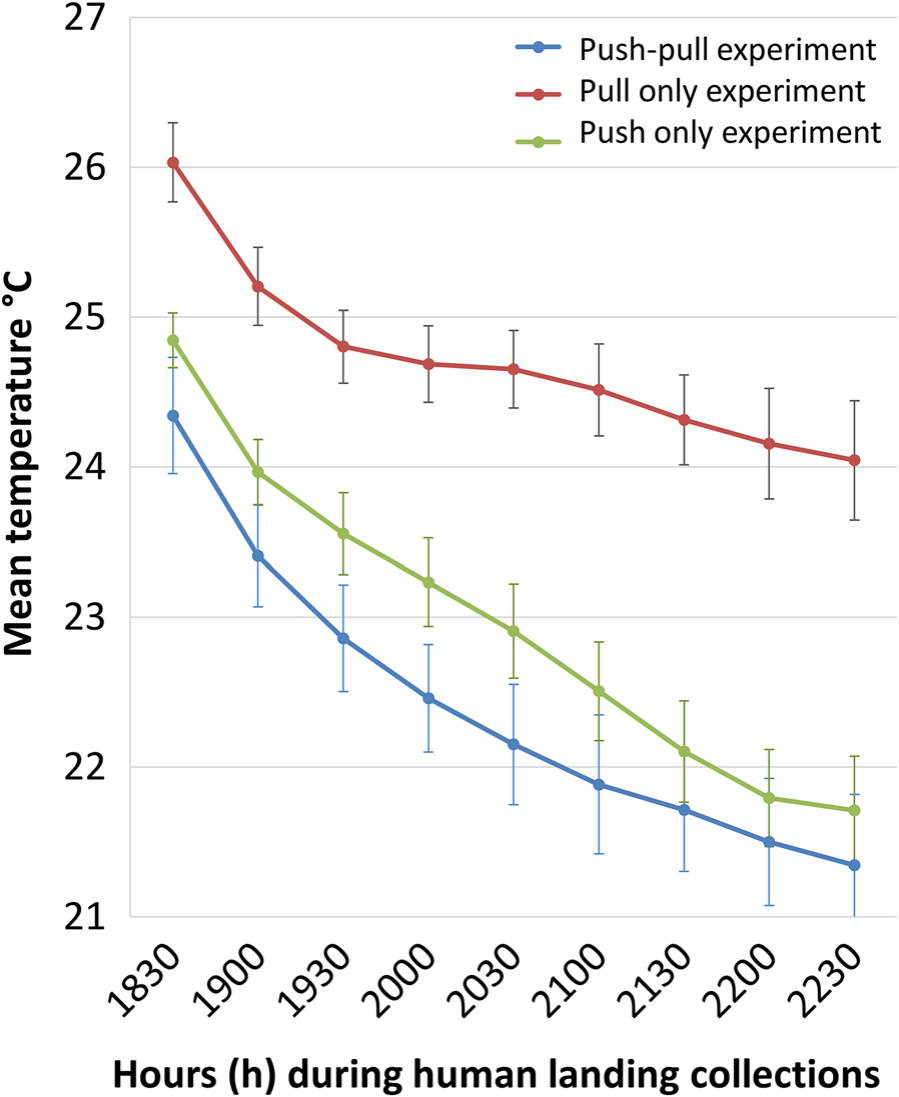
Hourly mean air temperature in the semi-field systems during the human landing collections (19.00–23.00 h) of different experiments. The data variability is shown with 95% confidence intervals

During all experimental set-ups, the temperature and relative humidity were logged to determine possible variations between experiments and the impact on experimental output. The mean temperature during the experimental hours for the baseline experiment (open eaves, no trap) was 23.6 °C (95% CI 23.4–23.7), while for the pull-only set-ups it was 24.7 °C (95% CI 24.6–24.8; Fig. 8). The mean temperature during the final push-pull experiment was with a mean of 22.2°C (95% CI 22.1–22.3) nearly 1° lower that during the push-only experiment with transfluthrin 23°C (95% CI 22.8–23.1; Fig. 8). Temperature during the Citriodiol^®^ experiments for the first concentration was 22.8 °C (95% CI 22.6–22.9) and 23.2 °C (95% CI 23–23.4) for the second concentration. The relative humidity was maintained at > 70% during the experimental hours throughout the different experimental set-ups.

### Detection of volatile chemicals released by the push-pull components

#### Air movement and temperature variations during chemical quantification

Air samples for chemical analysis were taken in August during the dry season and the average temperatures during the sampling hours were 20.7 °C (95% CI 20.4–21.0 °C). The air samplings were done in relatively still air. Air movement was recorded every 10 min during the sampling times and most of the recordings (75%) indicated ‘no movement’. During the remaining times a low air speed (0.6–1.7 m/s) was measured, consistently from a north-east to east-north-east direction (45°–66°). This meant, as indicated in Fig. 2, that at the sampling location the air moved from the direction of the hut towards the sampling points.

#### Detection and estimated concentration of transfluthrin

**Fig. 9:**
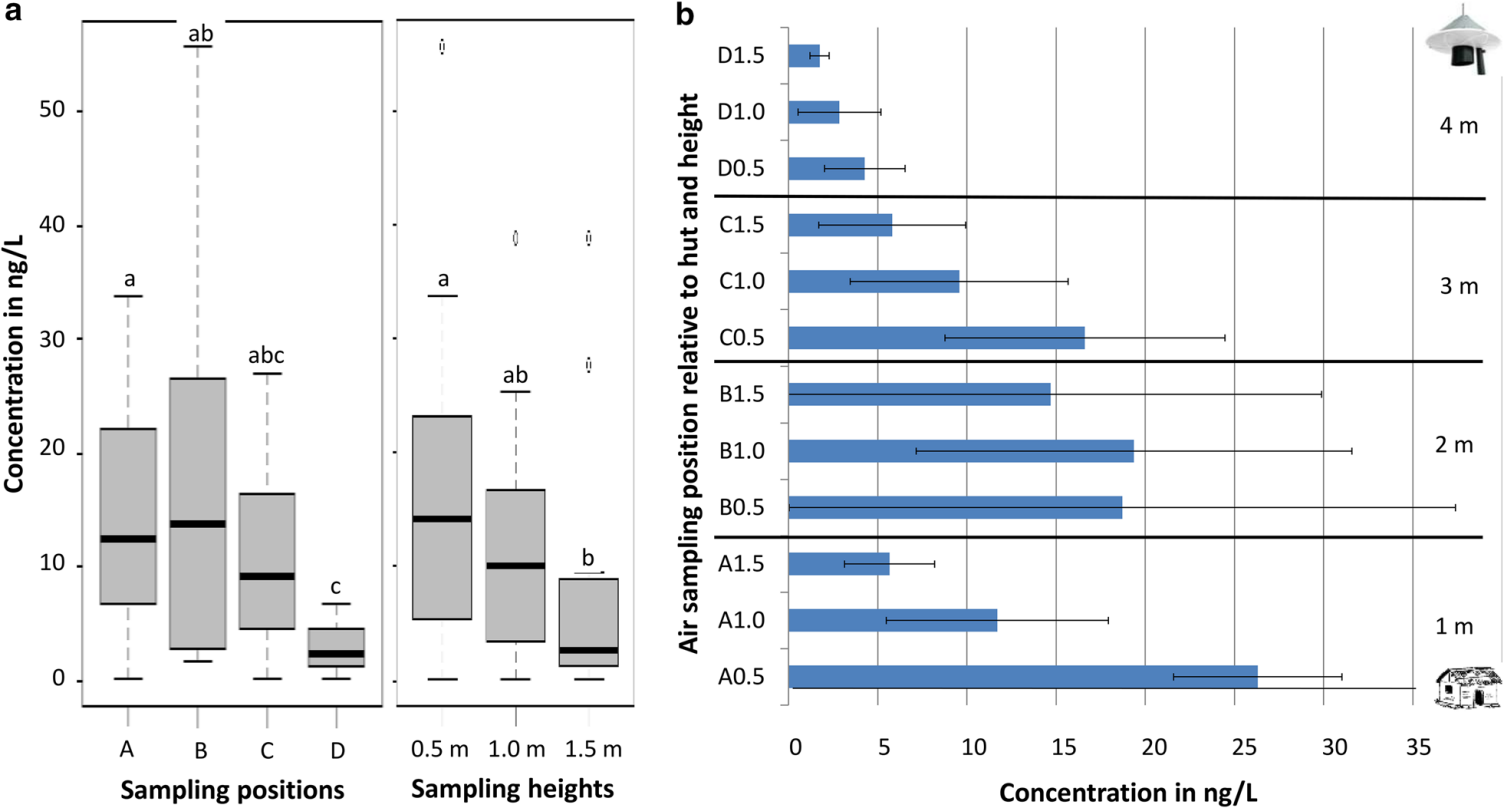
Transfluthrin concentrations estimated from air sampling at different distances and heights. Median concentrations in nanograms per litre of transfluthrin across the four sampling positions from A (1 m from release point) to position D (4 m from release point) as well as across sampling heights from 0.5m above the ground to 1.5 m (**a**); mean transfluthrin concentration (standard error bars) in nanograms per litre of air sampled for every sampling point (**b**)

Transfluthrin was detected at all sampling points. The concentrations decreased greatly with distance from the release point (positions A, B, C and D; Fig. 2) with the highest concentration detected nearest to the point of release (position A) and the lowest concentration being detected farthest away from the point of release (position D). Variations in concentrations at different heights were seen across all the sampling positions (*p* = 0.03) with a general trend for higher transfluthrin concentrations being found at lower sampling points of ≤ 1 m from the ground (Fig. 9). The averaged concentrations of transfluthrin at position A (nearest to experimental hut) and position D (nearest to Suna trap) were significantly different (*p* = 0.02; Fig. 9) as were the concentrations at positions B and D (*p* = 0.002; Fig. 9). The highest concentration of transfluthrin detected was 26.3 ng/l (95% CI 21.6°31.0 ng/l) at 1 m from the release point (position A) at 0.5 m from the ground. At 1.5 m of the same sampling position, the transfluthrin concentration was 5.7 ng/l (95% CI 3.1–8.2 ng/l). The lowest concentration was 1.7 ng/l (95% CI 1.2–2.3 ng/l), detected 4 m away from the release point (position D) 1.5 m above the ground.

#### Detection and estimated concentration of MB5 constituents

Two of the five MB5 constituents, namely l-(+)-lactic acid and ammonia solution, were not detectable under the analytical conditions since the stationary phase of the column used was for the detection of non-polar compounds while the two compounds are polar in nature [71, 72]. The remaining compounds, namely 3-methyl-1-butanol, butan-1-amine and tetradecanoic acid, were detected at low concentrations in some, but not all samples. Of the 60 samples collected, 3-methyl-1-butanol was quantified in only 15 (25%), with the rest falling below the detection limit. The highest concentration of 0.4 ng/l (95% CI 0.07–0.75 ng/l) was detected closest to the release point at position D, 1 m from the Suna trap. Contrasting to all other chemicals in the push-pull system, 3-methyl-1-butanol was found at the highest average concentration at 1.5 m above the ground. At the same position, the average concentration was 0.13 ng/l (95% CI 0–0.38 ng/l) at 1.0 m above ground and 0.04 ng/l (95% CI 0–0.13 ng/l) at 0.5 m.

**Fig. 10:**
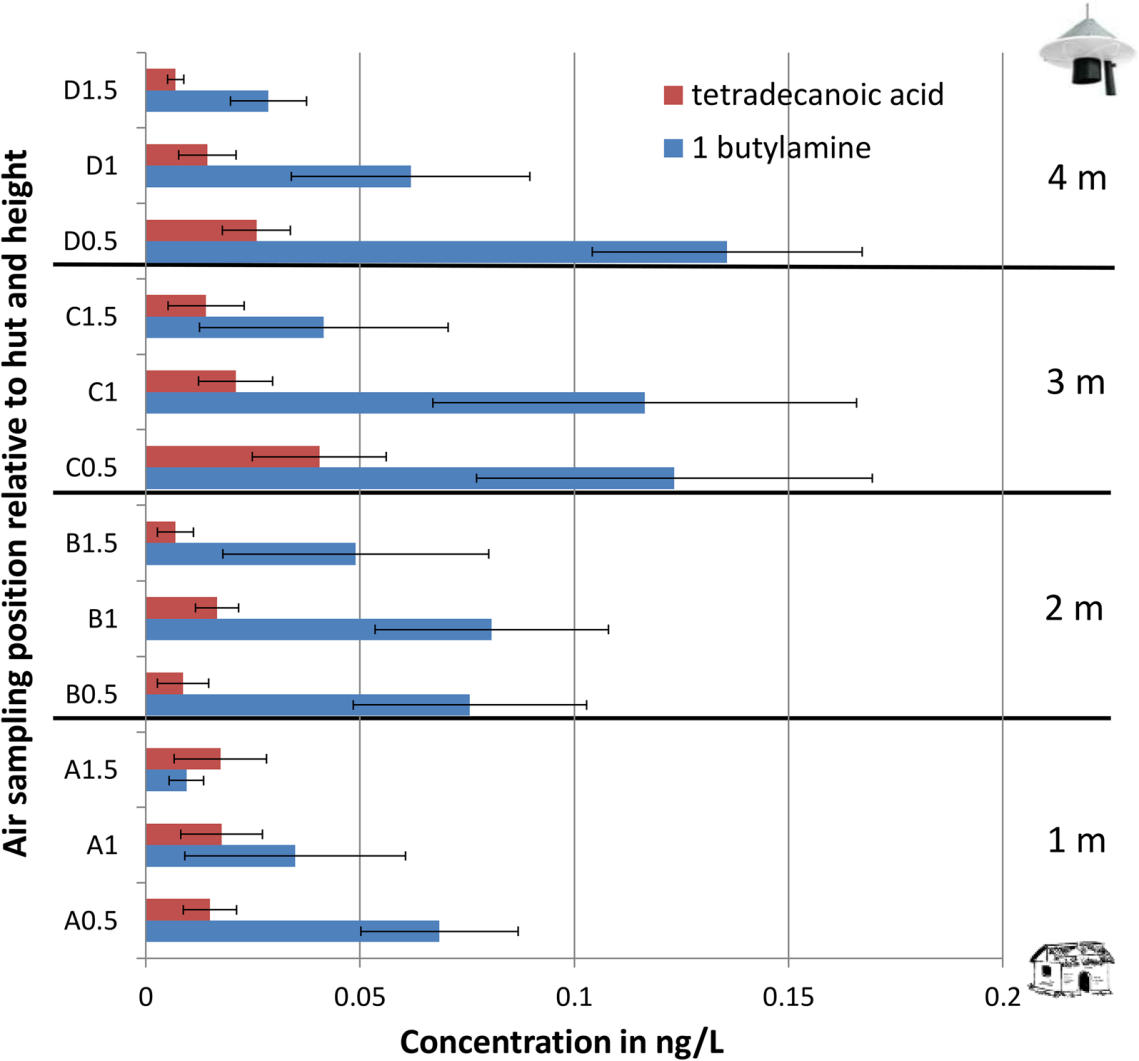
Average concentrations in nanograms per litre (standard error bars) of 1-butylamine and tetradecanoic acid across all air-sampling positions

Out of the 60 air samples, 1-butylamine was detected in 31 samples (52%), while tetradecanoic acid was detected in 42 samples (70% of samples). There was no strong association with distance and height for these chemicals, though some trends can be seen in Fig. 10. The chemical 1-butylamine was consistently detected at higher concentrations closer to the ground (≤ 1 m). This also applied to tetradecanoic acid, at least within 2 m from the release point (positions D and C). Generally, both chemicals were detected at significantly higher concentrations within 2 m of the releasing trap than further away.

## Discussion

The results of this study provide essential insight into the behaviour of host-seeking *An. arabiensis* in response to tools aimed at manipulating their odour orientation and consequently at reducing the number of potentially infectious bites in the peri-domestic area.

Transfluthrin-treated hessian fabric strips loosely fixed around eave gaps prevented, depending on the experimental conditions, between 40 and 80% of the *An. arabiensis* bites a human volunteer would have received in the absence of the treatment. On the contrary, the microencapsulated Citriodiol® did not show any spatial repellent properties as concluded from the unaffected human landing rates.

The components tested for pulling *An. arabiensis* vectors in an attract and kill approach did not perform to expectations based on previous work [50, 51, 54, 73–75]. The Suna trap baited with the MB5 odour blend and supplemented with CO_2_ from molasses fermentation attracted only a third of the released host-seeking females when no human host was in the vicinity, confirming similar studies [50, 62]. However, when a human volunteer was in the same system, the trap caught < 1% of all released mosquitoes whilst a human consistently attracted over two thirds of all mosquitoes released. This was the case in the absence (‘pull’ only) and in the presence of a spatial repellent (‘push-pull’ set-up). In its current configuration the tested ‘pull’ did not provide a valuable addition to a spatial repellent in a push-pull system for prevention of *An. arabiensis* bites. At closer scrutiny, in the absence of a human volunteer, it was found that the MB5 odour blend did not add much additional attraction to the trap, and it might be sufficient to only provide CO_2_ from molasses fermentation as bait. This confirms data shown graphically from field-based indoor trap collections of *An. arabiensis* [62] in western Kenya though the authors did not discuss this observation. Our experiments also clearly indicated that 2-butanone is not a suitable CO_2_ replacement for the collection of *An. arabiensis* confirming previous observations under similar experimental conditions [62].

Contrary to the majority of recent experimental studies with malaria vectors and odour-baited traps, here we included a human being in the system, since ultimately a push-pull intervention aims to directly protect people from bites and hence would require traps to compete well with the human host odour when placed in close vicinity. However, human beings remain more attractive to host-seeking malaria vectors despite all the efforts employed in identifying host-seeking cues and producing synthetic lures because of the high complexity in mosquito host-seeking behaviour [53, 76–78]. Our results support the findings of Okumu et al. [74] who observed that the chemical odour blend attracted host-seeking *An. arabiensis* in representative numbers in the absence of a human, but when presented with the two odour sources side by side within the same hut in field settings, the mosquitoes retained their preferences for humans. The authors suggested that preferences are dependent upon whether the stimuli are in direct short-range competition or whether they are far apart with completely separated odour plumes, which might be the best strategy to exploit for mass-trapping interventions [28, 79, 80].

Due to the increasing insecticide resistance levels in malaria vectors, this study aimed to explore Citriodiol^®^ with its active ingredient PMD as a spatial repellent since it belongs to a different class of chemicals than those currently used in public health. It is a well-known topical repellent [24, 25, 81–84] and has been suggested as having spatial repellent properties in a previous study [26]. However, the previous evaluation was done using electricity-powered active emanators to dispense the chemical. Furthermore, the product was not microencapsulated but applied as Citriodiol® oil at high concentrations on a nylon strip and several emanators used in a semi-field system less than a quarter of the size of those used here [26]. Such effort is neither operationally feasible nor cost-effective. Importantly, the previous study did not include a human blood host in the test system but used an MB5-baited trap as a substitute [26]. We opted for microencapsulation to secure the Citriodiol® into the fabric with the aim to allow passive slow-release of the repellent for possible long-term usage when fixed on eave gaps [85, 86]. However, neither of the two test concentrations resulted in any protection against mosquito bites, not even when the material was fixed very closely to the human volunteer on the chair (data not shown). Optimal formulation and presentation of a repellent, whether spatial or topical, is key for effectiveness [25] and further work might be warranted. For now, it remains unclear if the concentrations of chemicals released from the fabric were just too low or whether PMD does in fact not have spatial repellent properties.

Transfluthrin is a pyrethroid insecticide, which is not only known for its killing effect but also its moderate volatility, which makes it an effective spatial repellent [21, 87–89]. Transfluthrin has been incorporated into commercial products for mosquito control with encouraging outcomes [27, 40, 90–94]. Applied on hessian material for passive emanation, it has been proposed to protect from 70 to 90% of bites from Afrotropical malaria vectors in a range of experimental laboratory and field studies implemented in coastal and inland Tanzania [21, 49, 89, 95, 96]. Our results confirm the potential of transfluthrin for use as a spatial repellent vector control tool. However, the protective efficacy under most of our test conditions was much more moderate than in the Tanzanian studies. One reason for this might be the differences in average temperatures during experiments in the different regions [18, 49, 97–99]. During the implementation of our final push-pull experiment, only around 40% of the bites that would have been received without protection were averted. This was only half the protection we found for the 2.5 g/m^2^ transfluthrin treatment during the push-only experiment. Given that the pull-only experiments did not provide any evidence that the presence of the odour-baited trap might increase the proportion of mosquitoes attempting to bite the human volunteer, other factors are likely responsible for the lower protection from the spatial repellent at the time. Notably, during the push-pull experiment, evening temperatures were an average of 22 °C, around 1 °C lower than during the push-only experiment. Increases in temperature increase the effective vapour pressure of a chemical and therefore the volatilization rate [100]. Cooler temperature conditions lead to lower transfluthrin evaporation rates and it has been previously suggested that the protective efficacy of passively emanated transfluthrin from hessian fabric reduces when temperatures are < 23 °C [49]. Conversely, increasing temperatures were associated with increasing airborne transfluthrin concentrations in closed test systems [101] and an increase of mosquito mortality with an increase of airborne transfluthrin [22].

Our samples for quantification of transfluthrin in the air within 5 m from the release point were taken during the cold season with temperatures during sampling of around 21 °C. Nevertheless, the chemical was consistently detected with concentrations decreasing by an order of magnitude from > 20 ng/l to 1.7 ng/l over 5 m from the release point. These concentrations are significantly higher than those reported by Ogoma et al. [49] who reported 0.13 ng/l from samples collected indoors from a non-ventilated 30 m^3^ room; however, the treatment load of the hessian test material was also three times lower (0.8 g/m^2^) than in our study. Our estimated concentrations are, nonetheless, well below the maximum acceptable exposure concentration for long-term inhalation exposure of human beings of 500 ng/l, as defined by the regulatory authorities of the European Union [102]. Our findings relate well with a more recent study using a similar approach to ours on malaria vectors in Vietnam, where airborne transfluthrin concentrations were estimated at 1.32 ng/l at 4 m from the release point [22] and were below the detection limit further away. This study also showed higher knock-down and mortality rates for caged mosquitoes at ground level than above 1 m from the ground [22] supporting our observation of highest concentrations at 1.0 m and below. This might limit the ‘protective bubble’ especially in the outdoor environment around the house.

The inconsistent detection of the constituents of the putative attractant MB5 in the air might suggest that the odours were not sufficiently released and dispersed, specifically 3-methyl-1-butanol was rarely picked up by the adsorbent filters. Tetradecanoic acid and 1-butylamine were detected more frequently, though not consistently and at very low concentrations in close vicinity to the trap. Whether the chemical release rates and hence performance of the MB5-baited Suna trap might also be affected by nighttime temperatures during trapping needs further investigations. Mechanisms to increase the released concentrations and improve the dispersion might be explored in future by modifying the Suna traps [103] or surveying alternative traps and baits [29, 78, 104–106]. A recent study for example suggested the combination of transfluthrin treated fabric on eave gaps with BG Malaria traps (Biogents, Germany) and suggested a larger distance of the pull trap from the human host; nevertheless, the authors also found only a very marginal addition of protection from the traps in preventing *An. arabiensis* bites [95]. Strategies combining mass trapping of mosquitoes with spatial repellents at independent locations rather than combining on household levels should be explored in future.

Finding a pull component that is efficient enough to attract mosquitoes even when a human is close by, yet easy to set and maintain, remains desirable to remove adult vectors from the transmission setting. The idea to develop a push-pull system with the MB5-baited Suna trap was inspired by the successful mass-trapping field trial in western Kenya with the pull component only, which was associated with significant reductions in vector densities [28, 80]. However, when analysed by vector species, only *An. funestus* densities were reduced in the study site whilst *An. arabiensis,* which accounted for around a quarter of the vector population, were not affected, resulting in only a moderate reduction in malaria parasite prevalence in the study area [28, 80]. This suggests a species-specific attraction to the MB5-baited trap. Similar observations were made by Mburu et al. [62] when investigating the use of 2-butanone as a CO_2_ replacement in a rice irrigation area in western Kenya, where *An. funestus* is the predominant vector species.

## Conclusions

This detailed step-by-step evaluation of the selected putative repellent ‘push’ and attractive ‘pull’ components has led to a better understanding of their prospect to affect the host-seeking behaviour of the malaria vector *An. arabiensis* in the peri-domestic space and helps to gauge the impact such tools would have when used in the field for vector monitoring or control. The study has highlighted the need for testing odour-based interventions in the presence of a human host to gain accurate estimates. A trap cannot substitute a human being when changes in attraction and human landing rates are the outcome measure. Additionally, the importance of working with different vector systems has been elucidated. There is urgent need to further study potential differences in odour orientation between the two major vector species complexes: *An. gambiae* (*s.l.*) and *An. funestus* group. The here tested pull components, including the MB5 blend and 2-butanone presented in a Suna trap, have performed poorly and were ineffective in contributing to a functional push-pull vector control system. The search for highly efficient odour blends and suitable traps remains a research priority. Here it will be desirable to develop odour-baited traps that target all major vectors at the same time for use in varied eco-epidemiological systems. This study further confirmed that, at least under standardised experimental conditions, passive emanation of transfluthrin from treated hessian fabric strips around eave gaps can provide protection from mosquito bites in the peri-domestic space. Comparisons across published work have, however, also highlighted that the expected impact might be quite variable from location to location, depending on climate conditions and vector species. Data generated under standardized field conditions in a single location need to be interpreted in the local context and should be replicated under different conditions to ensure recommendations can be generalised or can be tailored to local contexts. Mathematical modelling should support decision making by integrating data from different settings in prediction models to understand the geographical range where such tool might be useful and the impact to be expected under a varying environmental and epidemiological conditions. Field evaluations are required to investigate how results from semi-field experiments correlate to findings from field trials.

For example, air movement was minimal in the semi-field systems and the repellent transfluthrin was detected within 5 m from the experimental hut. However, it must be assumed that this is quite different from natural conditions, especially during rainy seasons when vector densities and malaria transmission peak. Rainstorms characterising the tropical evenings might well interfere with the odour plumes and protective bubble around the house. One might therefore plausibly assume that the protective efficacy in the peri-domestic space in western Kenya field sites would be lower than the largely moderate effects observed in the current experiments.

## Data Availability

The datasets used and/or analysed during the current study are available from the corresponding author on reasonable request.

## Abbreviations

HLC: Human landing catches
GC-FID: Gas chromatography-flame ionization detector
